# Laparoscopic repair of an incarcerated diaphragmatic hernia after right hepatectomy for hepatic injury: a case report

**DOI:** 10.1186/s40792-018-0542-0

**Published:** 2018-11-19

**Authors:** Shohei Takaichi, Tsuyoshi Takahashi, Soichiro Funaki, Koji Tanaka, Yasuhiro Miyazaki, Tomoki Makino, Yukinori Kurokawa, Makoto Yamasaki, Kiyokazu Nakajima, Meinoshin Okumura, Masaki Mori, Yuichiro Doki

**Affiliations:** 1 Department of Gastroenterological Surgery, Osaka University Graduate School of Medicine, 2-2 Yamadaoka, Suita, Osaka 565-0871 Japan; 2 Department of respiratory Surgery, Osaka University Graduate School of Medicine, Osaka, Japan; 3 Toneyama National Hospital, 5-1-1 Toneyama, Toyonaka, Osaka 560-8552 Japan

**Keywords:** Diaphragmatic hernia, Right hepatectomy, Laparoscopic surgery

## Abstract

**Background:**

Diaphragmatic hernias (DH) are generally classified as either congenital or acquired. Acquired DH are generally of traumatic cause, being a rare complication after hepatectomy. Although repair of a DH can be performed via laparotomy, laparoscopy, or thoracoscopy, the use of laparoscopy is rare after hepatectomy owing to the formation of scar tissue. In this case, we describe our successful attempt at laparoscopic repair of a recurrent DH after hepatectomy.

**Case presentation:**

A 30-year-old man underwent right hepatectomy for trauma and thoracotomy via the eighth intercostal space, with direct closure of the diaphragm by suturing. The patient subsequently developed a right DH, with strangulation ileus of the small intestine. He underwent laparotomy 3 months after the initial surgery. The defect was observed to be clearly separate from the previously sutured area of the diaphragm. Five years after treatment, the patient developed abdominal pain and vomiting due to incarceration of the transverse colon in the right intrathoracic space (detected via abdominal computed tomography and radiography). The patient was again diagnosed with DH and underwent laparoscopic repair of the hernia with direct closure. The patient was discharged 11 days after surgery without further complication.

**Conclusions:**

A laparoscopic approach was feasibly and safely used to repair a recurrent DH after hepatectomy. The surgical approach will need to be decided in a patient-specific manner.

## Background

Diaphragmatic hernias (DH) are generally classified as either congenital or acquired. Acquired DHs are generally caused by trauma with the majority occurring on the left side [1], since the anatomical location of the liver minimizes the possibility of a right-sided hernia. DH is a rare complication following hepatectomy. After right-sided major hepatectomy, the large surface area of the right diaphragm, previously covered by the liver, may allow the right colon and small bowel to migrate into the infra-diaphragm fossa.

The first successful repair of a DH was performed in 1886 by Riolfi [2]. Currently, surgical repair of a DH can be performed by laparotomy, laparoscopy, or thoracoscopy. Although laparoscopic surgery has become a widely accepted technique, including for DH repair, a laparoscopic approach is usually not considered for repair of a recurrent DH after right hepatectomy because of the dense abdominal adhesion formation.

In this case, we report on our successful laparoscopic repair of a recurrent DH that developed 5 years after right hepatectomy.

## Case presentation

A 30-year-old man underwent right hepatectomy at previous hospital, due to trauma, in August 2012. At that time, a thoracotomy in the eighth intercostal space was performed for direct closure of the diaphragm. Pooling of right pleural effusion was treated with drainage up to postoperative day (POD) 9. The patient recovered and was discharged on POD 14. In November 2012, he presented with a right DH and strangulation of the ileus of the small intestine. He underwent laparotomy at the same previous hospital, found the defect which was separated from the previously sutured area of the diaphragm, and repaired it.

**Fig. 1:**
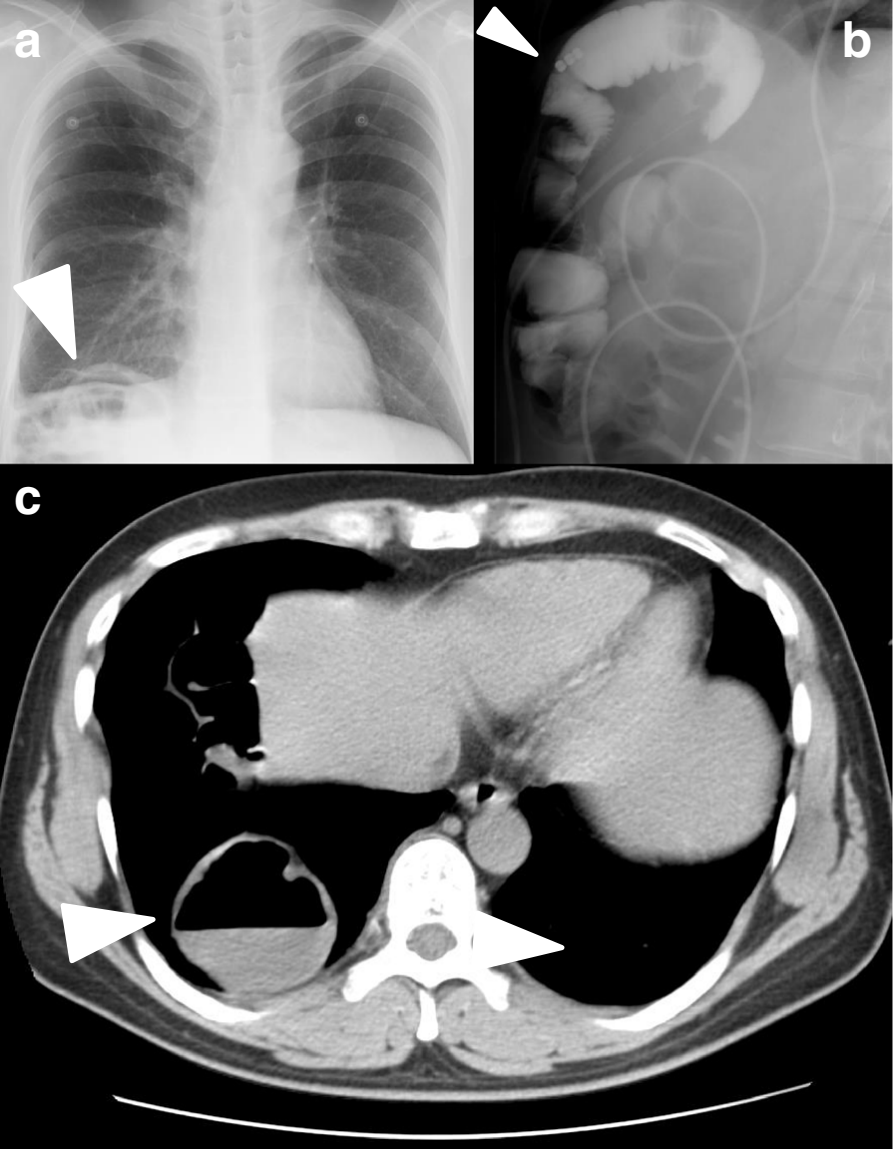
Preoperative examinations. Chest and abdominal plain radiographic findings. **a** The chest radiograph revealed intestinal air in the right lower lung field (arrow head). **b** The abdominal radiograph revealed dilation of the large intestine with gas (arrow head). **c** Computed tomography showing the herniated transverse colon located in the right thoracic cavity (arrow head)

In May 2017, the patient presented to the local hospital with abdominal pain and vomiting and was diagnosed with an intestinal obstruction; an ileus tube was inserted and the patient was referred to our hospital for treatment. Upon admission, the physical examination was unremarkable, and laboratory findings, including complete blood count, erythrocyte sedimentation rate, and biochemical tests, were all within normal limits. Chest and abdominal radiographs revealed colon gas in the right intrathoracic space (Fig. 1a, b). On contrast imaging, the ileus tube in the transverse colon was observed to be incarcerated in the right intrathoracic space (Fig. 1c), a finding which was confirmed on computed tomography (CT) of the chest and abdomen (Fig. 1d). The patient was diagnosed with a DH and underwent laparoscopic hernia repair.

**Fig. 2:**
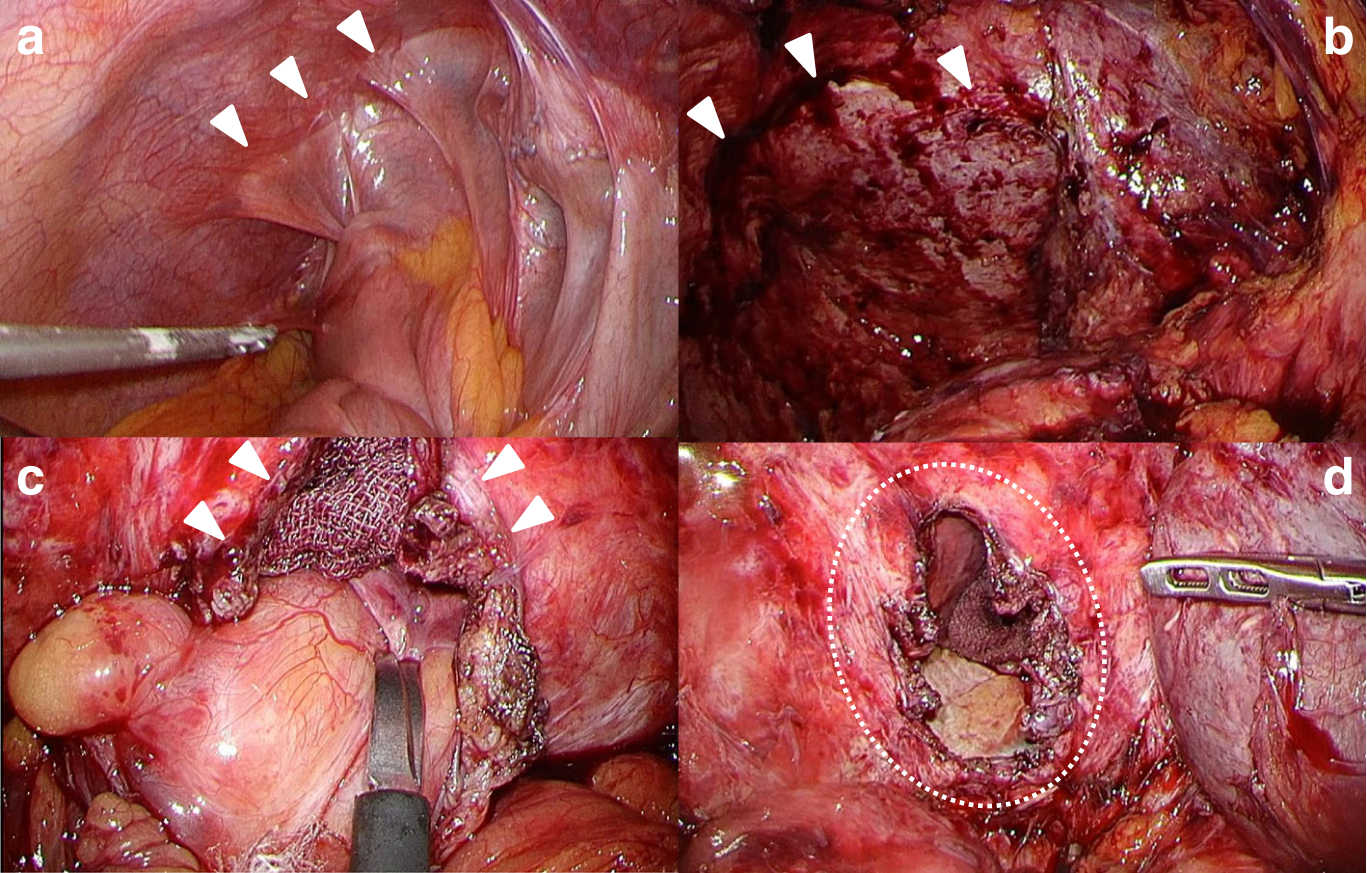
Operative findings. **a** The transverse colon and small intestine anastomosis were found to be adherent to the right hemi diaphragm (arrow heads). **b** The images show the surface of the liver with adhesions (arrow heads). **c** The transverse colon was incarcerated in the diaphragmatic defect (arrow heads). **d** We confirmed a 5 × 3 cm diaphragmatic defect by direct observation (circle)

**Fig. 3:**
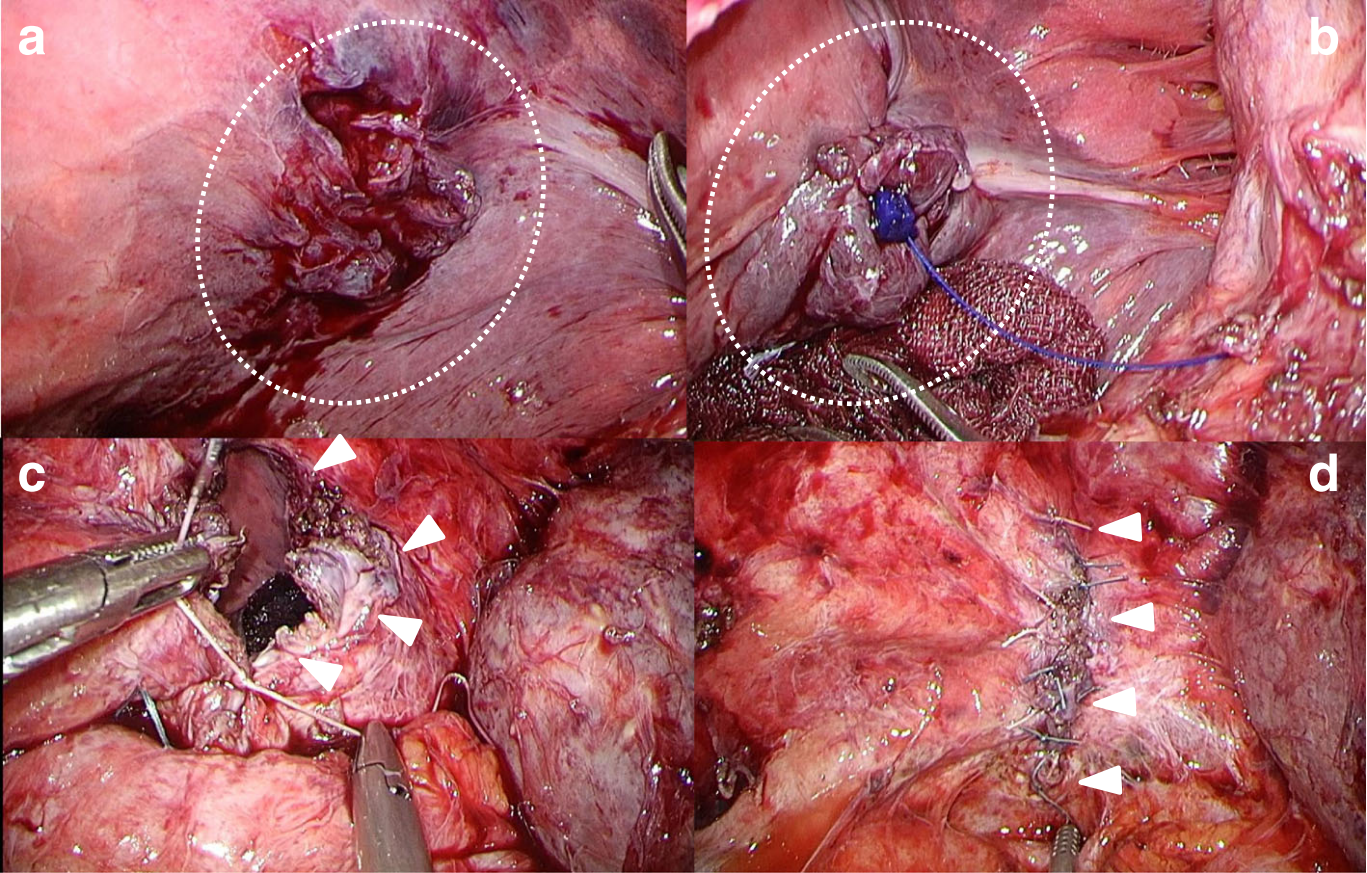
Operative findings. **a** The damaged pleura of the lung was peeled away from the transverse colon (circle). **b** Damaged pleural lining was repaired laparoscopically, using a trans-diaphragmatically (circle). **c** We closed the defect laparoscopically, using a non-absorbable 2–0 polyester suture (arrow heads). **d** Image of the repaired diaphragm (arrow heads)

The surgical repair was performed with the patient in the left half-lateral decubitus position. Four laparoscopic ports were placed in the abdomen as follows: a 12-mm port at the umbilicus for the scope, two 12-mm ports at the right lateral lesion as working ports, and a 5-mm port at the middle of the upper abdomen used as an assistant. Under laparoscopic view, the transverse colon and small intestine anastomosis were found to be adherent to the right hemidiaphragm (Fig. 2a), and we proceeded with careful separation (Fig. 2b). After dense adhesions between the herniated organs and the pleural lining of right lung were sectioned, 5 × 3 cm diaphragmatic defect was observed, with the transverse colon found to be incarcerated in the defect and adherent to right lung (Fig. 2c, d). We proceeded with widening the diaphragmatic defect, gently peeling the transverse colon from the lung, and repositioning the transverse colon into the abdominal cavity. The damaged pleural lining of the lung (Fig. 3a) was repaired by a respiratory surgeon using a laparoscopic transdiaphragmatic approach (Fig. 3b). The diaphragmatic defect was confirmed to be different from the area repaired previously. It was then closed laparoscopically with non-absorbable 2–0 polyester sutures (Fig. 3c, d). The postoperative course was uneventful, and a contrast study of the ileus tube demonstrated good passage, with no leakage or stenosis. At 6 months after the surgery, all symptoms had disappeared.

## Discussion

Diaphragmatic hernia is a rare complication after hepatectomy, with some case reports having been published and only two case series of treatment outcomes [3, 4]. Normally, the liver serves as a block against herniation of abdominal contents into the right thorax and, therefore, diaphragmatic hernia rarely occurs on the right side in persons without surgical history [5]. The clinical manifestation of diaphragmatic hernia can differ widely, depending on the side of involvement of the herniated organs. All DH that develop following right hepatectomy are right-sided [3, 4] which, in our case, included symptoms resulting from the entrapment of the ileus. Francesco et al. reported an incidence rate of postoperative DH following right-sided hepatectomy of 2.3% (3/131), with the DH development with a median delay of 14 months in their case series [3].

The risk factors for DH after hepatectomy include (1) undetected direct injury or thermal injury by electrocautery to the diaphragm during mobilization of the liver; (2) a fragile thin musculature in the congenitally weak area of the posterior diaphragm, between the costal and lumbar portions of the diaphragm; and (3) the pressure gradient between the abdominal and thoracic cavities. The defect may enlarge progressively, resulting from the constant diaphragmatic motion or the adhesions that form after surgery, with the inflamed postoperative scar tissue exerting traction on the diaphragm [6]. In addition, Scott et al. reported right hepatectomy for liver transplantation to carry the greatest risk for DH because of the right diaphragmatic attachments to the liver, which cover a relatively large surface area and are substantially more adherent than on the left side of the diaphragm and, therefore, increase the risk of inadvertent injury during liver mobilization [7]. The patient in our case report had undergone hepatectomy with a diaphragm incision and intra-operative direct repair, and then the defect of the DH was observed to be clearly separated from the suture area during the first laparotomy performed. Similarly, at the time of our DH repair, the defect was confirmed as being different from the suture area. In our patient, although the cause of DH was still unclear, thermal injury by electrocautery might have weakened the diaphragm during the previous procedure.

DH repair can be performed by laparotomy, laparoscopy, or thoracoscopy, with the approach selected based on the preference of a surgeon, the anatomical location of the defect, and the degree of sub-diaphragmatic adhesions. Tabrizian et al. reported that a thoracic approach might be the best approach when dealing with recurrence after an abdominal repair [4]. In our case, we selected a laparoscopic approach as it provided a better approach than thoracotomy for instrument and bowel manipulation, as well as being deemed to be more appropriate based on the expectation of adhesions in the thoracic due to prior thoracotomy. Moreover, the approach allowed us to separate the adhesion of the herniated bowel to the right of the diaphragm and to subsequently repair the damaged pleural lining using a laparoscopic transdiaphragmatic route.

Our review of the literature in PubMed, using “diaphragmatic hernia” and “hepatectomy” or “liver surgery” or “liver resection” as keywords, identified only two cases of DH after hepatectomy with both being repaired by laparoscopic procedure [4, 8]. We did not find reports of laparoscopic surgery for recurrent DH after hepatectomy. Therefore, our case is important in demonstrating the effectiveness of laparoscopic surgery for repair of a diaphragmatic defect, providing a sufficient working space and an increased field of view.

With regard to the repair of the defect itself, some authors have reported on the use of prosthetic material as reinforcement, whereas other authors preferred simple suturing of the defect. However, it is generally agreed that defects larger than 20–30 cm^2^ do require use of a prosthesis [9]. Different types of mesh can be used for DH repair, including polypropylene, composite, or biological mesh, with a polypropylene mesh being most commonly used. Although polypropylene mesh is more expensive than other types of mesh, it is preferred due to the low associated rate of infection [3]. In our case, the diaphragmatic defect was sufficiently small, allowing us to proceed with a direct suture repair and, thereby, avoiding adverse events associated with using a mesh.

In conclusion, in this rare case of recurrence of DH following hepatectomy for trauma, laparoscopic surgery was found to be effective for the diagnosis and repair of a DH.

## Abbreviations

CT: Computed tomography
DH: Diaphragmatic hernias
