# Correction to: Laparoscopic repair of an incarcerated diaphragmatic hernia after right hepatectomy for hepatic injury: a case report

**DOI:** 10.1186/s40792-018-0551-z

**Published:** 2018-12-21

**Authors:** Shohei Takaichi, Tsuyoshi Takahashi, Soichiro Funaki, Koji Tanaka, Yasuhiro Miyazaki, Tomoki Makino, Yukinori Kurokawa, Makoto Yamasaki, Kiyokazu Nakajima, Meinoshin Okumura, Masaki Mori, Yuichiro Doki

**Affiliations:** 1 Department of Gastroenterological Surgery, Osaka University Graduate School of Medicine, 2-2 Yamadaoka, Suita, Osaka 565-0871 Japan; 2 Department of respiratory Surgery, Osaka University Graduate School of Medicine, Osaka, Japan; 3 Toneyama National Hospital, 5-1-1 Toneyama, Toyonaka, Osaka 560-8552 Japan

**Correction to: Surg Case Rep**

**https://doi.org/10.1186/s40792-018-0542-0**

In the original publication of this article [1], the author found Fig. 1c has a redundant arrow. The correct Fig. 1c is below:

**Fig. 1:**
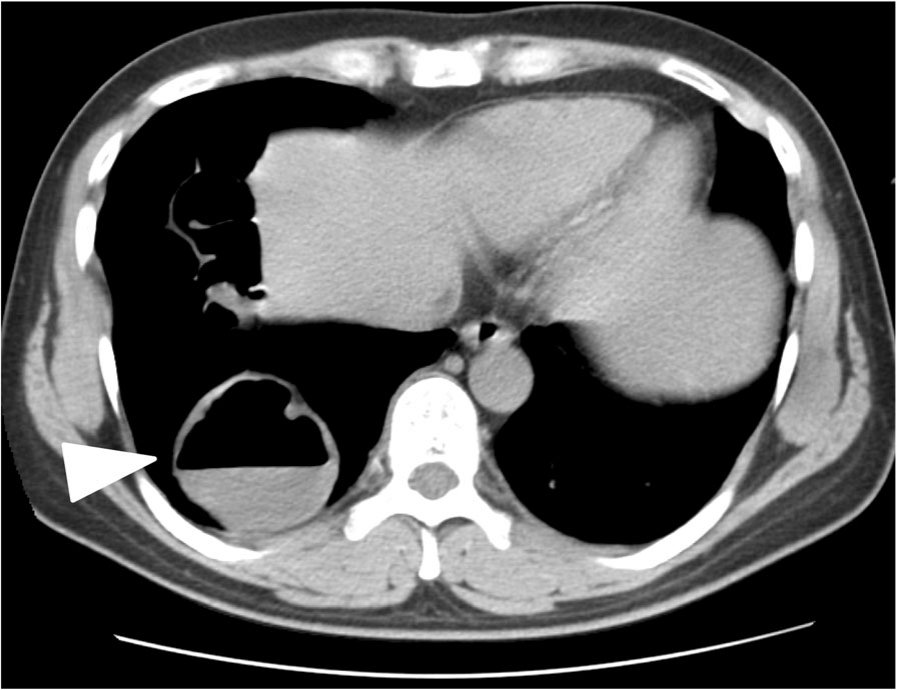
**c** Computed tomography showing the herniated transverse colon located in the right thoracic cavity (arrow head)
